# Effects of β-carotene intake on the risk of fracture: a Bayesian meta-analysis

**DOI:** 10.1186/s12891-020-03733-0

**Published:** 2020-10-31

**Authors:** Tesfaye Getachew Charkos, Yawen Liu, Kemal Sherefa Oumer, Ann M. Vuong, Shuman Yang

**Affiliations:** 1 Department of Epidemiology and Biostatistics, School of Public Health, Jilin University, Changchun, 130021 Jilin China; 2 Department of Epidemiology and Biostatistics, School of Public Health, University of Nevada, Las Vegas, USA

**Keywords:** Vitamin a, β-Carotene, Osteoporosis, Fracture, Bayesian, Meta-analysis

## Abstract

**Background:**

Epidemiological studies examining the association between β-carotene intake and risk of fracture have reported inconsistent findings. We conducted a meta-analysis to investigate the association between β-carotene intake and risk of fracture.

**Methods:**

We systematically searched PubMed, EMBASE and Cochrane library databases for relevant articles that were published until December 2019. We also identified studies from reference lists of articles identified from the clinical databases. The frequentist and Bayesian random-effects model was used to synthesize data.

**Results:**

Nine studies with a total of 190,545 men and women, with an average age of 59.8 years, were included in this meta-analysis. For β-carotene intake (1.76–14.30 mg/day), the pooled risk ratio (RR) of any fracture was 0.67 (95% Credible Interval (CrI): 0.51–0.82; heterogeneity: *P* = 0.66, I^2^ = 0.00%) and 0.63 (95%CrI: 0.44–0. 82) for hip fracture. By study design, the pooled RRs were 0.55 (95% CrI: 0.14–0.96) for case-control studies and 0.82 (95% CrI: 0.58–0.99) for cohort studies. By geographic region, the pooled RRs were 0.58 (95% CrI: 0.28–0.89), 0.86 (95% CrI: 0.35–0.1.37), and 0.91(95% CrI: 0.75–1.00) for studies conducted in China, the United States, and Europe, respectively. By sex, the pooled RRs were 0.88 (95% CrI: 0.73–0.99) for males and 0.76 (95% CrI, 0.44–1.07) for females. There was a 95% probability that β-carotene intake reduces risk of hip fracture and any type of fracture by more than 20%.

**Conclusions:**

The present meta-analysis suggests that β-carotene intake was inversely associated with fracture risk, which was consistently observed for case-control and cohort studies. Randomized controlled trials are warranted to confirm this relationship.

**Supplementary Information:**

The online version contains supplementary material available at 10.1186/s12891-020-03733-0.

## Background

Osteoporotic fractures are widely recognized as a major public health problem in the elderly [1, 2]. Approximately, the incidence of osteoporotic fracture affects 25% of females and 10% of males aged 60 years or above [3–5]. In 2000, there were an estimated 9 million osteoporotic fractures among individual’s ≥ 50 years worldwide, of which 1.6 million were hip, 1.7 million were forearm, and 1.4 million were clinical vertebral fractures [6]. Osteoporotic fractures are associated with an increased risk of mortality [7], chronic pain, loss of physical function, and ultimately decreased quality of life, financial burden, and psychosocial consequences, which significantly affect the individual as well as the family and community. As the average age of the world’s population continues to rise at an unprecedented rate, osteoporotic fractures will undoubtedly impact larger proportions of the population. Osteoporotic fractures have major implications among the aging population as it is associated with high morbidity and mortality [8, 9]. Annually, close to 65,000 deaths occurred due to complications of osteoporotic fractures [10]. In addition, osteoporotic fractures may result in functional loss and consequently disabilities, which further impose a considerable economic burden on society [8, 9, 11]. This impact is projected to increase over the next decades due to the increasing aging population [12].

Nutrition is an important modifiable factor influencing bone health [13]. Dietary intake of nutrients is a nonpharmacological intervention strategy for preventing the reduction of the loss of bone quality and the incidence of fractures. Several studies have investigated the effect of nutrition on bone health [13]. Fruit and vegetables are major sources of β-carotene antioxidants, which have bone health properties. A meta-analysis based on five prospective studies and two case-control studies reported that hip fracture risk decreased by 28% among participants with higher (vs. lower) dietary consumption of total carotenoids and β-carotene [14]. Carotene may reduce fracture risk by counteracting oxidative stress, which also can adversely affect bone mineral density [15–18].

The association between dietary β-carotene intake and fracture risk has been examined by several studies. Ambrosini et al. conducted a large cohort study that found a significant decrease in overall fracture risk (Relative Risk 0.89, 95% Confidence Interval 0.82–0.97) for β-carotene intake [19]. A study in China indicated that higher dietary intake of β-carotene was associated with lower risk of hip fracture in middle-aged and elderly adults, specifically with a 61% decreased in odds of hip fracture (95% CI 0.31, 0.49) [18]. Moreover, a number of studies consistently found that high β-carotene intake is associated with a protective relationship with fracture risk [17, 18, 20, 21], but not all [22–24]. Therefore, this meta-analysis aims to investigate the association between β-carotene intake and risk of fracture using a Bayesian hierarchical random effect model.

## Methods

The meta-analysis was conducted according to the Preferred Reporting Items for Systematic Reviews and Meta-Analyses (PRISMA) guidelines [25].

### Study selection

We systematically searched PubMed, EMBASE, and the Cochrane library databases for relevant studies that were published until December 2019. The medical subject headings (MeSH) used for the search were: “β-carotene” OR “Carotenoids” OR “Vitamin A” OR “Carotene” AND “Bone fracture” OR “Fracture” OR “Osteoporosis.” Reference lists from published articles identified from the clinical databases were also utilized to identify other relevant studies. Studies were included in this meta-analysis if they fulfilled the following criteria: (1) were written in the English language; (2) were original human studies; (3) had as the exposure of interest, β-carotene; (4) had as the outcome, fractures; and (5) provided risk estimates for the association between β-carotene and fractures.

### Data extraction

Two investigators (TGC and SY) independently extracted all relevant articles and identified eligible studies. During data evaluation, any disagreements were resolved through discussion. The following information were extracted from each included study: first author, publication year, country of origin, study design, the percent of women, mean age of the participants, risk ratios (RRs) and 95% CIs, fracture outcomes, exposure assessment methods, and the full list of covariate adjustments.

### Quality assessment

Newcastle-Ottawa Scale (NOS) was used to evaluate the quality of each individual study [26]. This scale assigns a maximum of nine stars to the following parameters: selection, comparability, exposure, and outcome. Studies with six star-items or less were considered as low quality, while those with at least seven star-items were considered as high quality.

### Statistical analysis

We converted the RRs to the logarithmic scale, and pooled these RRs using the DerSimonian and Laird random-effect models [27]; random effect models account for both within- and between- study variations. These results are presented in forest plots. Bayesian hierarchical models were used to perform the random-effects meta-analysis. We employed the Bayesian approach for its flexibility and ability to model a small number of studies [28]. It also accounts for the uncertainty of the parameters of interest, which is particularly important when data is sparse [29]. The probabilities of the exposure effect cannot be calculated with frequentist analyses since parameters of interest (i.e. RR) are treated as fixed. Moreover, Bayesian analyses allow prior information about the exposure effect to be incorporated with the current data (likelihood) to become the posterior distribution. The natural logarithmic of the RR follows a normal distribution with effect size (θ_*i*_) and within-study variance (δ_*i*_^2^). It is mandatory to specify prior distributions in the Bayesian Model. We applied three different prior distributions to the model. First, we applied the non-informative prior [30], which assigns equal likelihood on all possible values of the θ_*i*_ (i.e. we set RR equal to 1.0, with a large variance). The second prior was the skeptical prior distribution [31, 32], where we allowed only a 5% chance to observe a 10% risk change on fracture among β-carotene intake. Lastly, for the enthusiastic prior distribution, we assumed that β-carotene intake decreases 50% risk of fracture by half. A uniform distribution (0, 10) and an inverse gamma distribution (0.1, 0.001) were used for between-study variance (τ^2^).

In addition, subgroup analyses were performed based on study design, geographical region of the study population, sex, and by the site of fracture. Heterogeneity across studies was assessed using Cochran’s Q-statistic test and inconsistency was quantified by the I^2^ statistic [33]. The Egger’s tests were performed to identify any possible evidence of publication bias [34]. All analyses were performed using the WinBUGS program (Version 1.4.3, MRC Biostatistics Unit, Cambridge, UK) and the R program (Version: 3.4.3; R Foundation for Statistical Computing, Vienna, Austria).

## Results

### Study characteristics

**Fig. 1:**
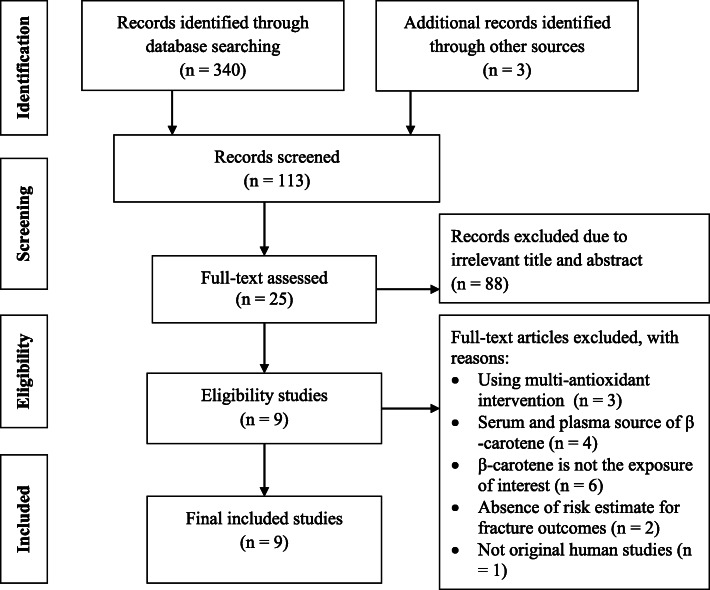
Flow chart for study inclusion and exclusion

A flow chart summarizing the process of study selection is shown in Fig. 1. A total of 343 articles were identified from the electronic database search. Of these, 230 articles were excluded due to duplicates and unrelated titles. After screening, 88 articles were excluded based on titles and abstracts that were irrelevant to our study aim. Finally, 9 articles with a total of 190,545 men and women were included in the final analysis. Out of these, three studies were performed in the United States (US) [17, 18, 24], one in Australia [19], three in China or Singapore [20, 21, 35], one in the Netherlands [36], and one in the United Kingdom [37] (Table 1). The participants’ age was in the range of 25 to 90 years (average age: 59.8 ± 10.2 years). NOS score ratings for the 9 studies ranged from 5 to 9-stars, with seven studies scoring 7+ stars. These seven studies were considered as high-quality based on their NOS score. The NOS scores for the 9 included studies in the present meta-analysis are shown in Table 1.

**Table 1:** Characteristics of included studies examining the association between β-carotene intake and risk of fractures

Author, year	Study design	Sample size	Percent of Women	Fracture outcomes	Mean age	Exposure assessment	Covariate adjustment^a^	Country	NOS score	Items that did not earn a NOS score
Feskanich, 2002 [24]	Cohort	72,337	100	Hip fracture	60	FFQ: Self-reported	1, 7, 8, 14, 15, 16, 17, 18, 19, 20	USA	7	- Study controls for any additional factor - Complete follow up, all subjects accounted for
Zhang, 2006 [18]	C-C	2564	69.2	Hip fracture	75.2	FFQ: Self-reported	1, 2, 8, 9, 11, 12, 14, 18, 19, 21	USA	9	
Sahni, 2009 [17]	Cohort	1046	61	Hip fracture	75	FFQ: Self-reported	1, 2, 8, 9, 12, 14, 19, 21, 22	USA	8	- Truly representative of the average sample in the community
Ambrosini, 2013 [19]	Cohort	2322	28.6	Any fracture	55	Medical records	1, 2, 6, 8, 14, 23	Australia	5	- Truly representative of the average sample in the community - Independent blind assessment - Study controls for any additional factor - Complete follow up, all subjects accounted for
Sun, 2014 [20]	C-C	1452	NA	Hip fracture	70.5	FFQ: Self-reported	1,2, 3, 4, 5, 6, 8, 9, 10, 11, 12, 13, 14	China	7	- Consecutive or representative series of cases - Community controls
Dai, 2014 [23]	Cohort	63,154	55.8	Hip fracture	56.3	FFQ: Self-reported	1, 3, 8, 9, 11, 12, 14, 15, 24, 25, 26, 27, 28	Singapore/China	7	- Complete follow up, all subjects accounted for - Independent blind assessment
Jonge, 2015 [22]	Cohort	5288	58.9	Any fracture	67	FFQ: Self-reported	1, 2, 3, 5, 8, 9, 11, 14, 29	Netherland	8	- Selected an adequate follow up period for outcome of interest
Hayhoe, 2017 [37]	Cohort	40,242	55.3	Any/hip/spine fracture	60.8	7-day food diaries	1, 6, 7, 8, 9, 11, 12, 14, 15, 30, 31	UK	9	
Cao, 2018 [21]	C-C	2140	74.1	Hip fracture	70.6	FFQ: Self-reported	1, 3, 4, 5, 6, 7, 8, 10, 11, 12, 14, 32	China	5	- Consecutive or representative series of cases - Study controls for any additional factor - Same method of ascertainment for cases and controls - Same non-response rate for both groups

*Abbreviations*: *C-C* case-control, *FFQ* food frequency questionnaire, *NA* not available, *UK* United Kingdomm, *US* United States,

^a^Age (1), sex (2), educational level (3), occupation (4), household income (5), family history of fracture (6), smoking status (7), alcohol intake (8), calcium use (9), multivitamin supplement use (10), physical activity (11), daily energy intake (12), dietary intake of selected nutrients (13), body mass index (14), use of postmenopausal hormones (15), hours of leisure-time activity (16), use of thiazide diuretics (17), protein use (18), vitamin D (19), vitamin K (20), caffeine use (21), height (22), medication use (23), dialect group (24), vitamin B6 (25), soy isoflavones (26), history of diabetes and stroke (27), use of hormone replacement therapy (28), disability index (29), hormone replacement therapy (30), corticosteroid use (31), and calcium supplement use (32)

### Association between β-carotene intake and fracture risk

**Fig. 2:**
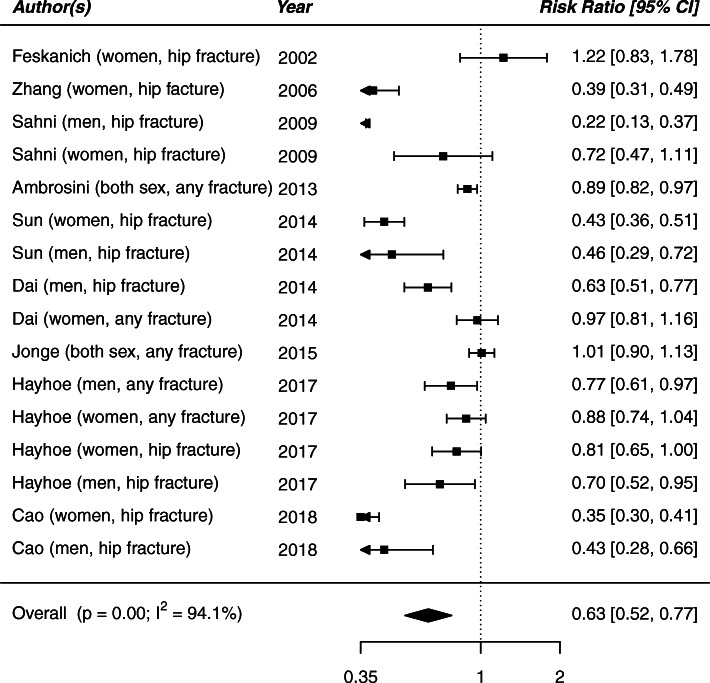
Forest plot of β-carotene intake and risk of fracture for all studies utilizing the traditional meta-analysis

The observed RR and 95% CI of each study are shown in Fig. 2. In the traditional meta-analysis approach, β-carotene intake (1.76–14.30 mg/day) was negatively associated with fracture risk (RR 0.63, 95% CI: 0.52–0.77). Statistically significant heterogeneity was observed for fracture risk across all included studies (*P* < 0.001, I^2^ = 94.1%).

Under the skeptical prior, β-carotene intake was associated with a 12% decrease in the risk of fracture (RR 0.88; 95% Credible Interval [CrI] 0.76–0.98). Inverse associations were also noted using the posterior probability distribution, specifically there was a 95% probability that β-carotene intake reduces the risk of any fracture by at least 20% (Table 2). A negative association between β-carotene intake and risk of hip fracture was found (RR 0.63; 95% CrI: 0.44–0.82), with a significant heterogeneity across studies (*p* < 001, I^2^ = 91.8%; Figure S1).

**Table 2:** Association between β-carotene intake and risk of fracture under the Bayesian meta-analysis

Subgroup	No. of studies	RR (95% CrI)	Probability (%) that the RR is:		
			≤ 1.0	≤ 0.9	≤ 0.8
Overall studies	9	0.67 (0.51, 0.82)	1.00	0.99	0.95
Hip fracture	6	0.63 (0.44, 0.82)	0.99	0.99	0.95
By study design					
Case-control studies	3	0.55 (0.14, 0.96)	0.95	0.92	0.88
Cohort studies	6	0.82 (0.58, 0.99)	0.92	0.77	0.45
By Geographic region					
US	3	0.86 (0.35, 1.37)	0.75	0.62	0.46
Europe	3	0.91 (0.75, 1.00)	0.91	0.44	0.10
China/ Singapore	3	0.58 (0.28, 0.89)	0.97	0.95	0.91
By Sex					
Females	8	0.76 (0.44, 1.07)	0.92	0.83	0.66
Males	6	0.88 (0.73, 0.99)	0.91	0.85	0.75

*Abreviation*: *RR* risk ratio, *95% CrI* 95% credible interval

### Subgroup analyses

**Fig. 3:**
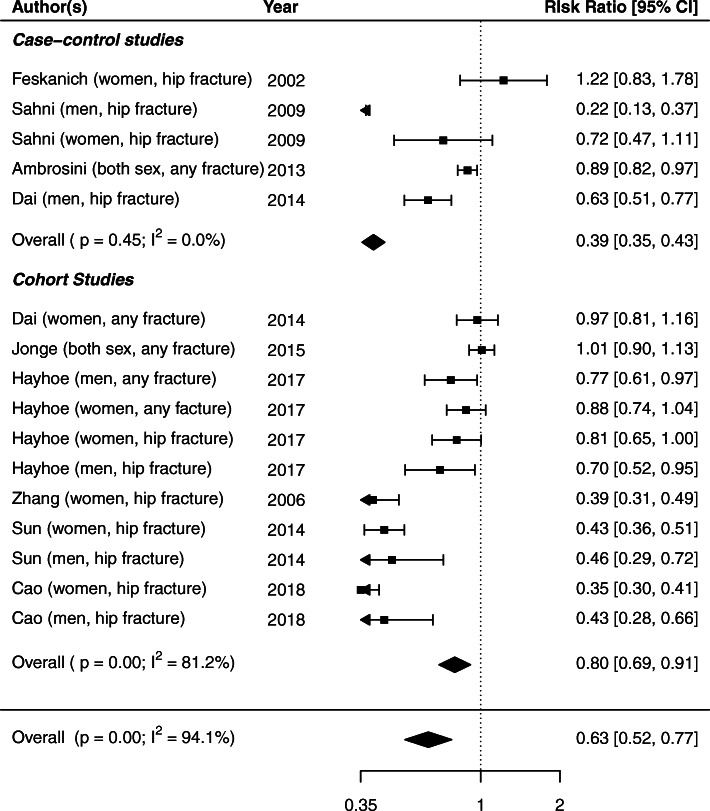
Forest plot of associations between β-carotene intake and risk of fractures under the traditional meta-analysis method, by case-control and cohort studies

In subgroup analyses, the pooled RR for the association between β-carotene and fracture risk was 0.82 (95% CrI: 0.58–0.99) in cohort studies and 0.55 (95% CrI: 0.14–0.96) in case-control studies, suggesting protective associations (Table 2). Statistically significant evidence of heterogeneity was found in cohort studies (*p* < 001, I^2^ = 81.2%), but not in case-control studies (*p* = 0.45, I^2^ = 0.0%; Fig. 3). By geographic region, the pooled RR between β-carotene and fracture risk was 0.58 (95% CrI: 0.28–0.89) for studies conducted in China/Singapore, 0.86 (95% CrI: 0.35–0.1.37) for studies in the US, and 0.91 (95% CrI: 0.75–1.00) for studies in Europe. Evidence of heterogeneity was observed across studies conducted in the US (*p* < 001, I^2^ = 92.2%) and in China/Singapore (*p* < 001, I^2^ = 97.3%), but not in Europe (*p* = 0.09, I^2^ = 47.7%; Figure S2). Subgroup analysis by sex resulted in a pooled RR of 0.88 (95% CrI: 0.73–0.99) for males and 0.76 (95% CrI: 0.44–1.07) for females for the association between β-carotene and fracture risk, which may indicate that β-carotene’s role in improving bone health may benefit females slightly more than males. Heterogeneity was observed for both sexes (Males: *p* < 001, I^2^ = 79.4%; Females: *p* < 001, I^2^ = 95.0%; Figure S3).

### Publication bias

**Fig. 4:**
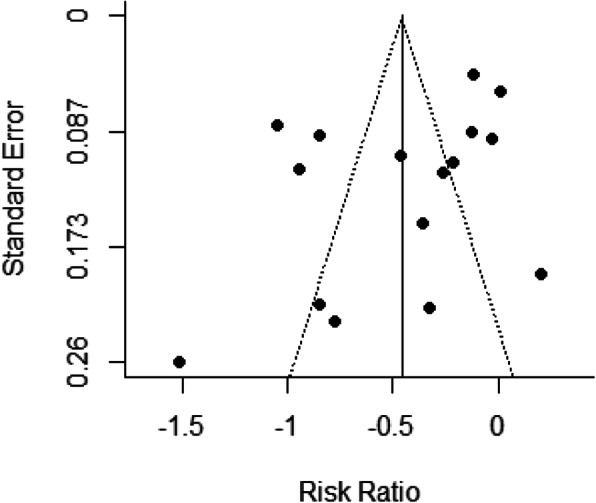
Funnel plot of publication bias, risk ratio versus standard error

We did not observe asymmetry across the studies (Fig. 4). No significant evidence of publication bias was found using Egger’s test (*P* = 0.09) and Begg’s test (*P* = 0.19).

## Discussion

In this meta-analysis, we investigated the association between β-carotene intake and the risk of fractures utilizing 9 peer-reviewed studies consisting of 190,545 men and women. We found that dietary β-carotene intake (1.76–14.30 mg/day) was associated with a 12% reduction in risk of fractures. In addition, higher intake of β-carotene was associated with lower risk of hip fractures. The findings of our meta-analysis suggests that higher dietary intake of β-carotene may have a favorable role in the protection of fracture risk.

To our knowledge, this is the first meta-analysis that synthesizes the relationship between β-carotene intakes, derived solely from dietary sources, with the risk of fractures. Our findings were consistent with the results of a previous meta-analysis published by Xu et al. [14] that concluded high intake of dietary β-carotene significantly decreased the risk of hip fracture by 28% (OR 0.72; 95% CI: 0.54–0.95). However, findings from the present meta-analysis were discordant with the recently published meta-analysis by Zhang et al. [38], which observed that higher β-carotene intake was weakly associated with increased risk of total fracture (RR 1.07; 95% CI: 0.97, 1.17), though the results were not statistically significant. The differences in the findings between our study and Zhang et al.’s may be due to the lower number of studies included by Zhang et al. [38]. Further, Zhang et al. [39] did not differentiate between the assessment measures of β-carotene, including serum, plasma, and dietary intake of β-carotene in their analysis, while our study focused solely on dietary β-carotene measures.

In the current meta-analysis, we also found an inverse association between β-carotene intake and risk of fracture across both prospective cohort and case-control studies. This may strengthen the robustness of our results. Regarding sex, we found a lower risk of fracture for females compared to males among high (vs low) β-carotene intakes. This may be a plausible result given hormonal differences between sexes. These results provide additional information beyond those published by the two meta-analyses [14, 38] that reported a null association between β-carotene intake and fracture risk in females, but not in males. Differing conclusions in sub-analyses by geographic region for the relationship between dietary β-carotene and factures are likely due to variations in the study populations, specifically with regard to genetics, diverse dietary habits that may be tailored to each culture, and lifestyle factors.

The underlying mechanism for the association between β-carotene intake and lower incidence of fracture risk remains unclear. However, some probable biological mechanisms have been proposed. A sufficient intake of vitamin A, including β-carotene, is essential for normal physiological activities [40] by affecting the growth hormone axis [41, 42]. Although, some evidence from animal studies suggest that antioxidant β-carotene contributes to the body’s defense against reactive oxygen species [43]. Thus, oxidative stress is thought to play an important role in the development of several chronic diseases, including osteoporotic fracture. Therefore, antioxidant β-carotene may have a beneficial effect against oxidative stress related to osteoporosis. β-carotene enhances osteoclastogenesis and reduces osteoblast apoptosis by stabilizing the β-catenin signaling pathway, which leads to a decrease in bone resorption [44–46]. In addition, carotenoids may interfere with growth factor receptor signaling by regulating IGF-1/IGFBP3, which is associated with cognitive function [47]. Impaired cognitive function is a known risk factor for falls and hip fractures [48].

There are some limitations in our meta-analysis. First, the β-carotene intake consumption level is not consistent between the identified studies. In addition, fruit and vegetable consumption patterns among countries are quite different. This might influence the reliability of our results. Second, the methods of β-carotene intake assessment across studies varied. Some studies assessed β-carotene using a validated food frequency questionnaire, while others used a questionnaire that was not. Third, the extracted RR was adjusted for differing sets of confounders between studies. Further, some of the potential confounding factors (i.e., age, physical activity, supplementary carotenoid intake, smoking, and vitamins) were not taken into account, which contributes to heterogeneity and sparse finding between individual studies. Lastly, our analysis was based on observational studies, which cannot determine a causal relationship between β-carotene and fractures.

## Conclusions

The present meta-analysis generated a pooled-RR using a novel Bayesian approach to assess the association between β-carotene intake and risk of fracture utilizing 9 peer-reviewed observational studies. We found that β-carotene intake was inversely associated with fracture risk, which was consistently observed in both case control and cohort studies. The observed findings support the role of β-carotene as a potential protective factor for fractures. High intake of fruit and vegetables that are rich in β-carotene antioxidants may be beneficial for bone health and may reduce the risk of fractures. It is recommended that randomized controlled trials are conducted to confirm the potential protective relationship we observed between β-carotene intake and fractures.

## Supplementary Information

**Additional file 1: Figure S1.** Forest plot of β-carotene intake and risk of hip fractures under the traditional meta-analysis approach

**Additional file 2: Figure S2.** Forest plot of the association between β-carotene intake and risk of fracture under the traditional meta-analysis approach, stratified analysis by geographic region

**Additional file 3: Figure S3.** Forest plot of observational studies examining the association between β-carotene intake and risk of fractures utilizing the traditional meta-analysis approach, stratified by sex

## Data Availability

The datasets used and/ or analyzed during the current study are available from the corresponding author on reasonable request.
