# Supplementary material for: Effects of β-carotene intake on the risk of fracture: a Bayesian meta-analysis
Source: BMC Musculoskelet Disord. 2020 Oct 31;21:711. doi: 10.1186/s12891-020-03733-0 (PMC7603770; doi:10.1186/s12891-020-03733-0)

**Supplementary figure**

**Fig.S1** Forest plot of β-carotene intake and risk of hip fractures under the traditional meta-analysis approach.


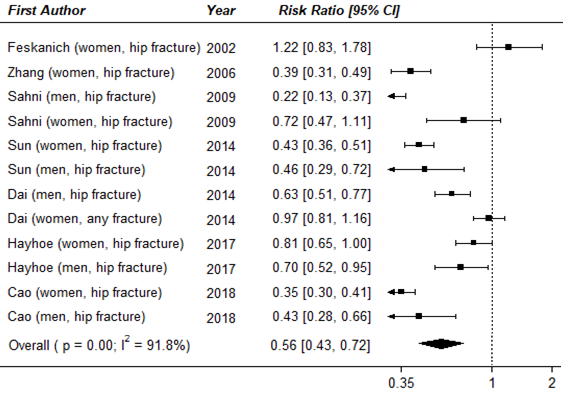

Supplement: Supplementary file 1 — Additional file 1: Figure S1. Forest plot of β-carotene intake and risk of hip fractures under the traditional meta-analysis approach [file 12891_2020_3733_MOESM1_ESM.docx]
