# Supplementary material for: Effects of β-carotene intake on the risk of fracture: a Bayesian meta-analysis
Source: BMC Musculoskelet Disord. 2020 Oct 31;21:711. doi: 10.1186/s12891-020-03733-0 (PMC7603770; doi:10.1186/s12891-020-03733-0)
Supplement: Supplementary file 2 — Additional file 2: Figure S2. Forest plot of the association between β-carotene intake and risk of fracture under the traditional meta-analysis approach, stratified analysis by geographic region [file 12891_2020_3733_MOESM2_ESM.docx]

**Fig.S2** Forest plot of the association between β-carotene intake and risk of fracture under the traditional meta-analysis approach, stratified analysis by geographic region.

**
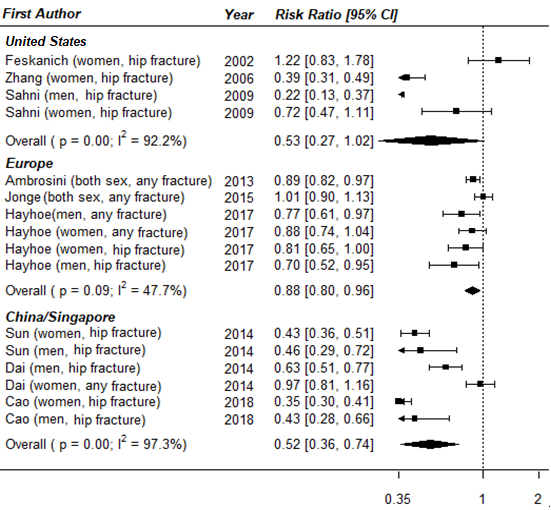
**
