# Supplementary material for: Effects of β-carotene intake on the risk of fracture: a Bayesian meta-analysis
Source: BMC Musculoskelet Disord. 2020 Oct 31;21:711. doi: 10.1186/s12891-020-03733-0 (PMC7603770; doi:10.1186/s12891-020-03733-0)

**Fig. S3** Forest plot of observational studies examining the association between β-carotene intake and risk of fractures utilizing the traditional meta-analysis approach, stratified by sex.


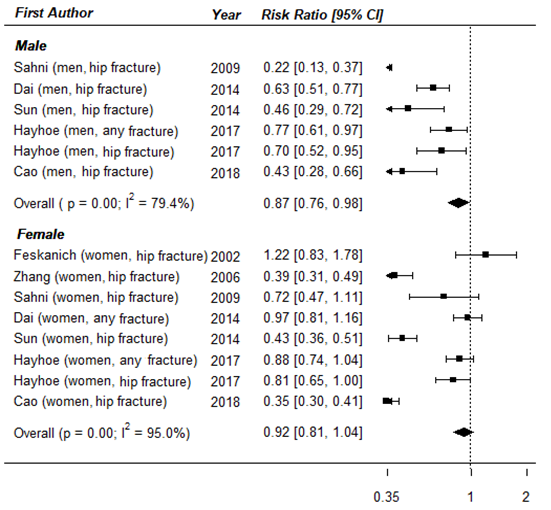

Supplement: Supplementary file 3 — Additional file 3: Figure S3. Forest plot of observational studies examining the association between β-carotene intake and risk of fractures utilizing the traditional meta-analysis approach, stratified by sex [file 12891_2020_3733_MOESM3_ESM.docx]
